# Regulation of SETD2 stability is important for the fidelity of H3K36me3 deposition

**DOI:** 10.1186/s13072-020-00362-8

**Published:** 2020-10-06

**Authors:** Saikat Bhattacharya, Jerry L. Workman

**Affiliations:** Stowers Institute for Medical Research, Kansas City, MO 64110 USA

**Keywords:** Chromatin, Proteasome, Histone, Methylation, Aggregation

## Abstract

**Background:**

The histone H3K36me3 mark regulates transcription elongation, pre-mRNA splicing, DNA methylation, and DNA damage repair. However, knowledge of the regulation of the enzyme SETD2, which deposits this functionally important mark, is very limited.

**Results:**

Here, we show that the poorly characterized N-terminal region of SETD2 plays a determining role in regulating the stability of SETD2. This stretch of 1–1403 amino acids contributes to the robust degradation of SETD2 by the proteasome. Besides, the SETD2 protein is aggregate prone and forms insoluble bodies in nuclei especially upon proteasome inhibition. Removal of the N-terminal segment results in the stabilization of SETD2 and leads to a marked increase in global H3K36me3 which, uncharacteristically, happens in a Pol II-independent manner.

**Conclusion:**

The functionally uncharacterized N-terminal segment of SETD2 regulates its half-life to maintain the requisite cellular amount of the protein. The absence of SETD2 proteolysis results in a Pol II-independent H3K36me3 deposition and protein aggregation.

## Background

The N-terminal tails of histones protrude from the nucleosome and are hotspots for the occurrence of a variety of post-translational modifications (PTMs) that play key roles in regulating epigenetic processes. H3K36me3 is one such important functionally characterized PTM. In yeast, this mark suppresses cryptic transcription from within the coding region of genes by preventing histone exchange [1]. In mammalian cells, it is involved in the recruitment of DNA repair machinery, in splicing and also, in establishing DNA methylation patterns by acting as a binding site for the enzyme DNMT3a [2–6]. Recent reports have emphasized the tumor-suppressive role of H3K36me3 in renal cancer especially, where the gene coding for the SETD2 histone methyltransferase is often deleted or mutated [7–9].

In yeast, the SET domain-containing protein Set2 (ySet2) is the sole H3K36 methyltransferase [10]. ySet2 interacts with the large subunit of the RNA polymerase II, Rpb1, through its SRI (**S**et2–**R**pb1 **I**nteraction) domain, and co-transcriptionally deposits H3K36me3 [11]. The deletion of the SRI domain from ySet2 abolishes both the Set2–RNA Pol II interaction and H3K36me3 methylation in yeast [12]. H3K36 methylation is a highly conserved histone mark and Set2 homologs are found in more complex eukaryotes [13]. These homologs share the conserved features like the AWS (**a**ssociated **w**ith **S**ET), SET [**S**u(var)3–9, **E**nhancer-of-zeste and **T**rithorax] and Post-SET domains that are required for the catalytic activity of the enzyme, and also, the protein–protein interaction domains such as the WW and the SRI. Notably, the mammalian homolog, SETD2, has a long N-terminal segment that is not present in ySet2. The function of this region has remained obscure [13, 14].

Here, we show that SETD2 is an inherently aggregate-prone protein and its N-terminal region regulates its half-life. This, in turn, is important for the fidelity of the deposition of the functionally important H3K36me3 mark. Our findings reveal that the previously uncharacterized N-terminal region of SETD2 is important in governing appropriate SETD2 function and activity.

## Results

### SETD2 is robustly degraded by the ubiquitin–proteasome pathway

**Fig. 1:**
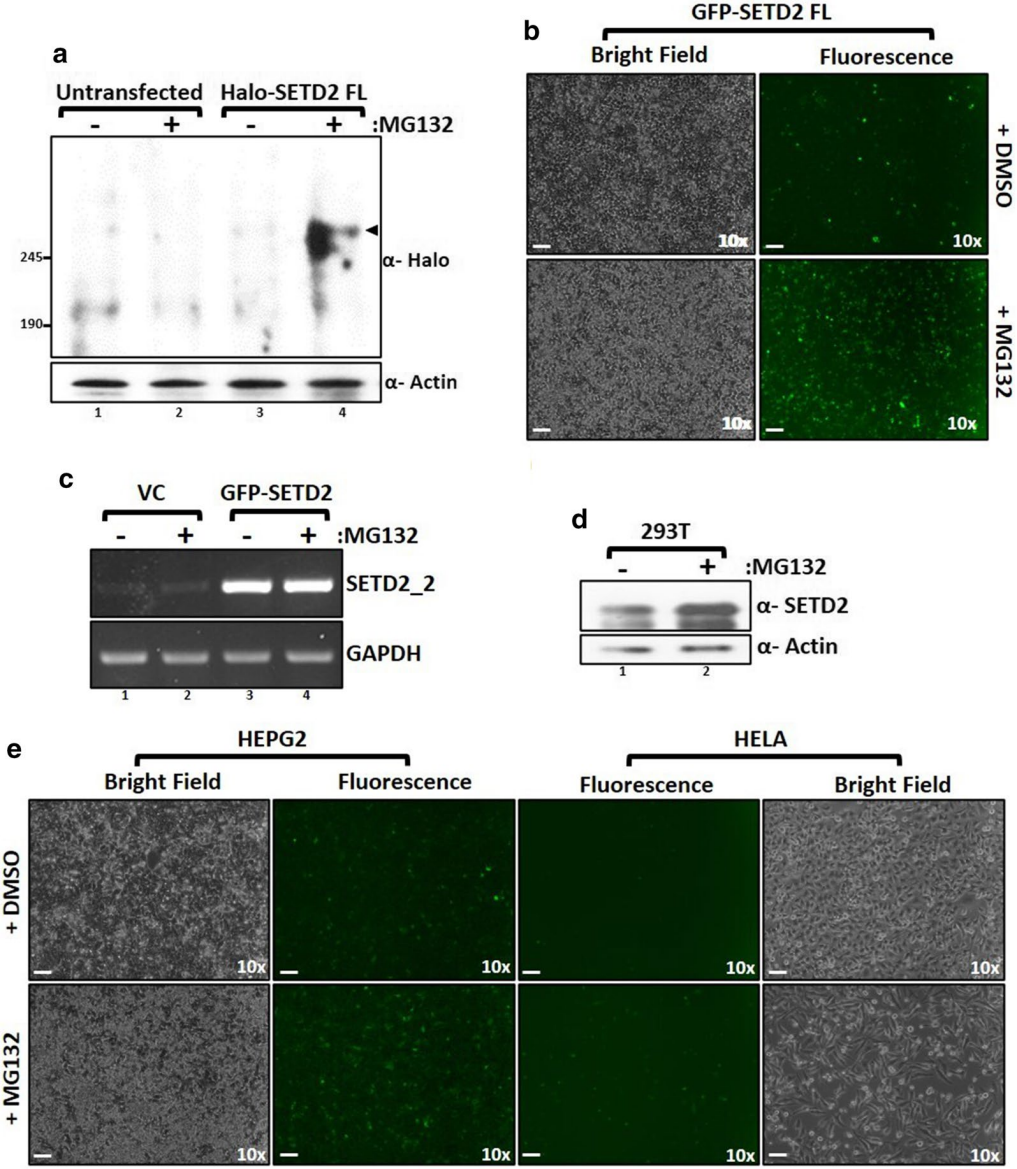
SETD2 is robustly degraded by the proteasome. **a** Western blot of whole-cell lysates probed with the depicted antibodies. Lysates of wild type 293T (untransfected) cells or expressing SETD2 full-length (FL) were prepared after 12 h of MG132 (10 µM) treatment. The expected band for the target protein is indicated by an arrow. **b** Microscopy images showing the effect of MG132 treatment on expression of GFP-SETD2 FL in 293T cells. The scale bar is 1 mm. **c** RNA was isolated from GFP-SETD2 FL transfected cells and RT-PCR was performed to check the transcript levels. GAPDH was used as a normalization control. VC- empty vector control. **d** Whole-cell lysates of 293T cells were prepared after 12 h of MG132 (10 µM) treatment and probed with the antibodies depicted. **e** Microscopy images showing the effect of MG132 treatment on the expression of GFP-SETD2 FL in the cell lines described. The scale bar is 1 mm

To understand the regulation of the SETD2 enzyme in human cells, we introduced a construct to express Halo- or GFP-tagged SETD2 FL (full length) under the control of the CMV promoter in 293T cells. Strikingly, the expression of GFP-SETD2 FL was barely detected post-72 h of transfection although the expression of GFP in vector control transfected cells was robust (Additional file 1: Figure S1a). Also, RT-PCR revealed a marked increase in the transcript level of SETD2 suggesting a robust transcription from the constructs introduced (Additional file 1: Figure S1b). Similar results were obtained with Halo-SETD2 FL transfected cells in which the expression was not detected with the western blotting of whole-cell lysates with an anti-Halo antibody (Fig. 1a, lane 3).

Autophagy and the ubiquitin–proteasome **s**ystem (UPS) are the major pathways for protein degradation in mammalian cells [15]. To investigate whether autophagy plays a role in SETD2 turn-over, 293T cells expressing GFP-SETD2 FL were treated with increasing concentration of the lysosome inhibitor, chloroquine. Chloroquine treatment did not have an apparent effect on the GFP-SETD2 FL protein level (Additional file 1: Figure S1c).

To test whether SETD2 is targeted for degradation by the UPS, SETD2 FL expression was checked after treating the cells with the proteasome inhibitor MG132. Proteasome inhibition led to an increase in the accumulation of SETD2. This was confirmed by western blotting of whole-cell lysate of Halo-SETD2 FL-expressing cells as well as microscopy visualization of GFP-SETD2 FL expression (Fig. 1a, b). The addition of MG132 did not have a prominent effect on Halo or GFP expression (Additional file 1: Figure S1d, e). Also, RT-PCR did not reveal any change in the transcript abundance of both endogenous and exogenous SETD2 upon MG132 treatment, suggesting that the increased SETD2 protein abundance observed is due to protein stabilization (Fig. 1c). Importantly, an increase in the accumulation of the endogenous SETD2 was also observed upon MG132 treatment of WT 293T cells, implying that SETD2 degradation by the proteasome is not limited to the recombinant form of the protein (Fig. 1d). Degradation of SETD2 leads to the appearance of lower molecular weight bands on a denaturing gel. Hence, the specificity of the SETD2 antibody was validated (Additional file 1: Figure S2). Next, the expression of GFP-SETD2 FL was tested in HEPG2 and HELA cells. SETD2 behaved similarly in these cell lines. Very weak expression of GFP-SETD2 FL was observed which increased upon proteasome inhibition by MG132 treatment (Fig. 1e).

Our results suggest that the short half-life of SETD2 is due to UPS-mediated decay which is consistent with previous findings [16]. Also, this mechanism for SETD2 proteolysis is not cell line specific.

### The removal of N-terminus region stabilizes SETD2

**Fig. 2:**
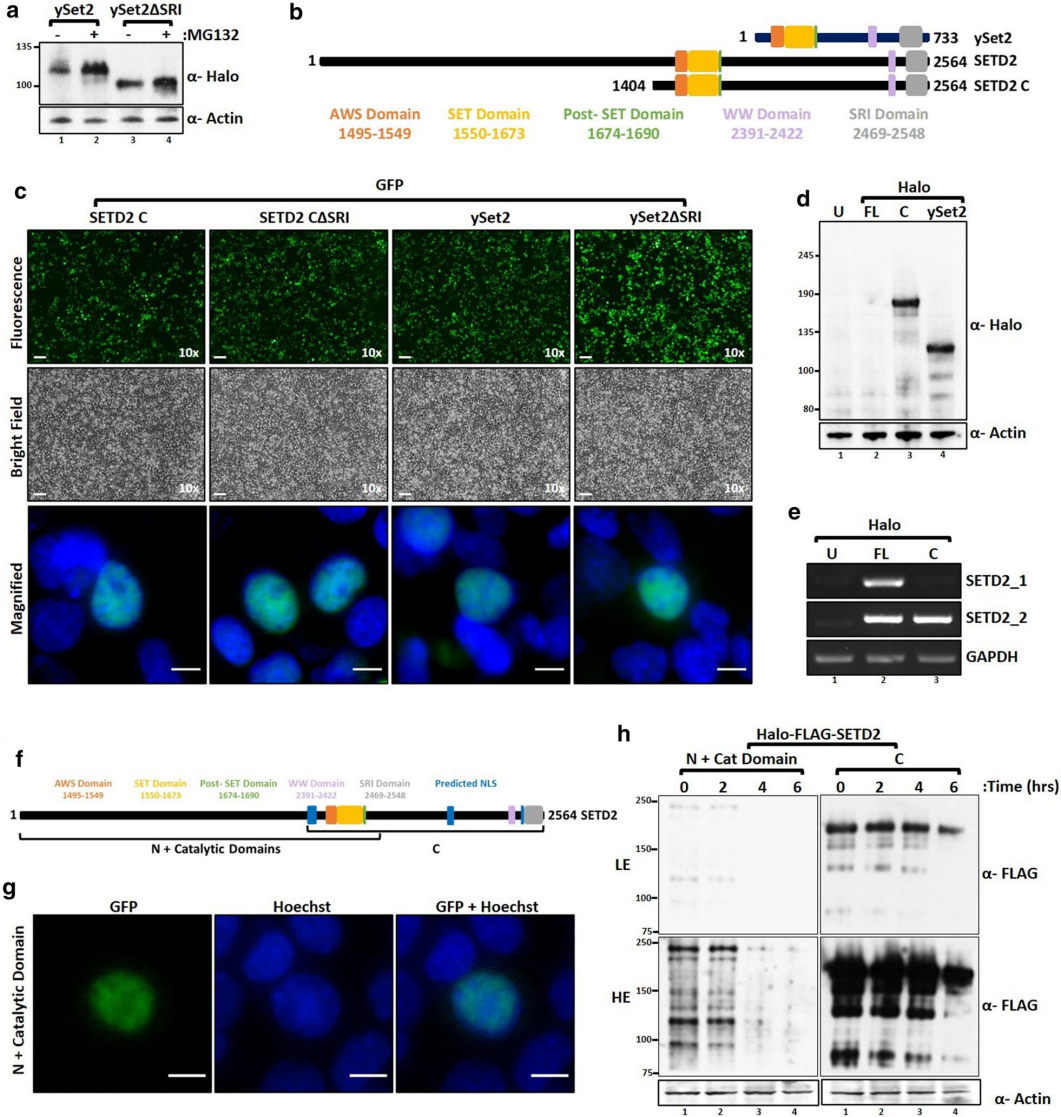
Removal of the N-terminal region of SETD2 leads to its stabilization. **a**, **d** Western blot of whole-cell lysates probed with the depicted antibodies. Lysates of 293T cells expressing Halo-ySet2 constructs were prepared after 12 h of MG132 (10 µM) treatment. U-untransfected. **b** Cartoon illustrating the known domains of yeast Set2 (ySet2), SETD2, and the SETD2 C-terminus region that shares the conserved domains with ySet2. **c** Microscopy images showing the expression and localization of GFP-SETD2 C and ySet2. The scale bar is 1 mm for 10 × images and 10 µm for the magnified ones. **e** RNA was isolated from transfected cells described in **d** and RT-PCR was performed to check the SETD2 transcript levels. GAPDH was used as a normalization control. SETD2_1 primer pair binds to the N-terminal segment of SETD2 transcripts, whereas, SETD2_2 binds to the C-terminal region which is common in both SETD2 FL and C. **f** Cartoon illustrating the SETD2 segments used in the cycloheximide-chase experiment. **g** Microscopy images showing the expression and localization of the GFP-SETD2 N + Catalytic Domain of SETD2. The scale bar is 10 µm. **h** Western blot of whole-cell lysates probed with the depicted antibodies. Lysates of 293T cells expressing Halo-FLAG-SETD2 constructs were prepared after cycloheximide (10 µg/ml) treatment. The duration of the treatment is shown in the figure. HE- High Exposure, LE-Low Exposure

Previously, the expression of yeast Set2 (ySet2) has been used in human cells to rescue H3K36me3 [17]. Hence, we next investigated the expression of Halo-ySet2 in 293T cells. Western blotting of whole-cell lysate with an anti-Halo antibody revealed a robust expression of ySet2 (Fig. 2a). ySet2 is a well-characterized protein that is degraded by the proteasome in yeast [18]. The UPS and the architecture of the proteasome itself are conserved from yeast to mammals [19, 20]. Consistent with that, our data show that ySet2 responds to MG132 treatment in 293T cells (Fig. 2a). This suggests that ySet2 is targeted for degradation through the UPS in human cells too, although, its expression level is much higher than that of SETD2 FL. SETD2 has an N-terminal region which is absent in ySet2 (Fig. 2b). We speculated that the disparity in the expression between ySet2 and SETD2 FL could be due to the presence of this segment in SETD2.

For this, we tested the expression level of a truncated mutant of SETD2, SETD2 C. Consistent with our hypothesis, microscopy of cells expressing the GFP-tagged proteins revealed that the expression level of SETD2 C was comparable to that of ySet2 (Fig. 2c). Analysis of whole-cell lysate with anti-Halo western blotting revealed that the expression of SETD2 C was markedly higher than SETD2 FL and was comparable to ySet2 (Fig. 2d). RT-PCR using primers specific for SETD2 FL and SETD2 C transcripts confirmed that the transcripts were produced robustly (Fig. 2e). Also, the transcript levels of SETD2 FL and SETD2 C were comparable.

To ascertain that the differences in protein levels observed were indeed due to the differences in protein half-lives, we performed a cycloheximide-chase experiment. For this, two overlapping segments of SETD2 were used (Fig. 2f). The catalytic domain of SETD2 was included in both the segments in case the SETD2 methyltransferase activity is important for its half-life. Microscopy with the GFP-tagged version of the protein revealed that the N-terminal + Catalytic domain of SETD2 was nuclear (Fig. 2g). Next, 293T cells expressing the Halo-FLAG-tagged N-terminal + Catalytic domain of SETD2 or SETD2 C were treated with cycloheximide to inhibit protein translation. Whole-cell extracts were prepared at different time-points and the expression level of SETD2 was scored by probing with an anti-FLAG antibody. The protein level of the N + Catalytic Domain segment was barely detectable 4-h post-treatment though SETD2 C still remains abundant (Fig. 2h). This suggests that the difference in protein half-lives that arises upon the removal of the N-terminal region of SETD2 is a significant contributor towards the greater accumulation of SETD2 C.

Collectively, the results suggest that the proteolytic instability of the full-length SETD2 might be a key contributor to the differences observed in protein abundance between SETD2 FL and SETD2 C.

### Unlike the full-length protein, SETD2 fragments express robustly

**Fig. 3:**
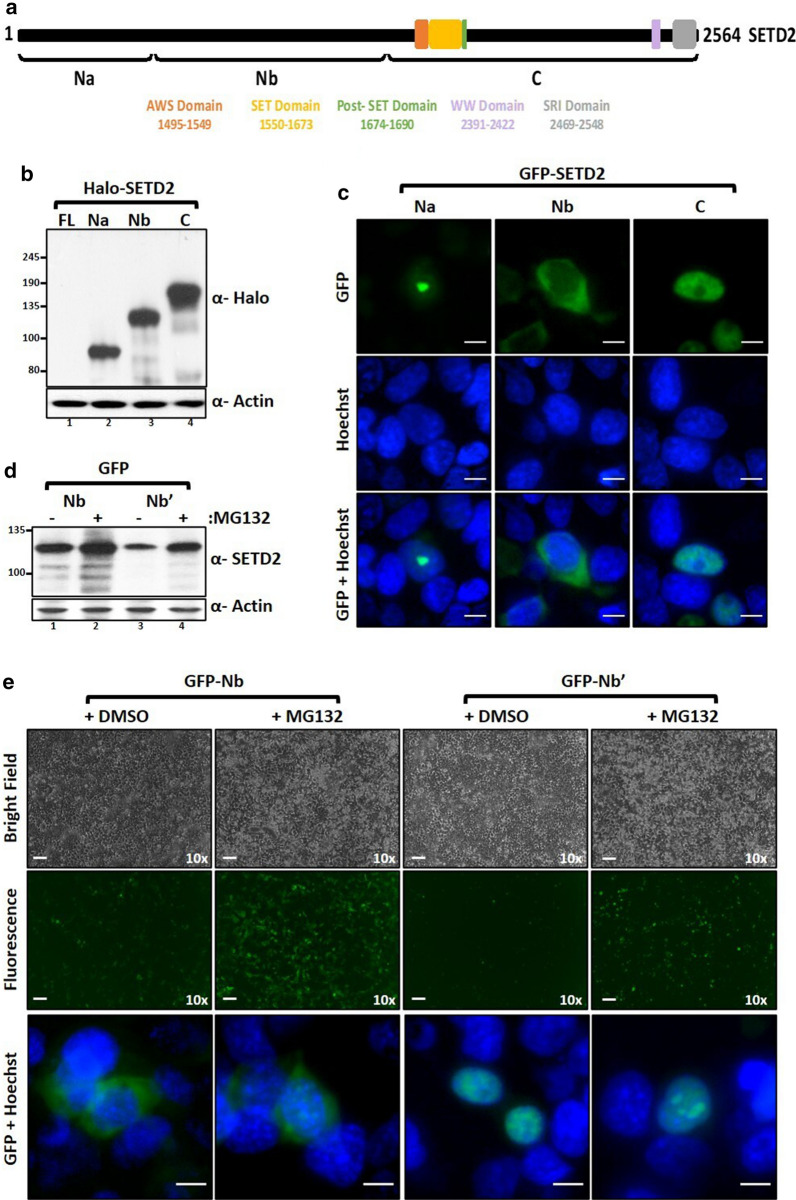
SETD2 fragments express robustly. **a** Cartoon illustrating the fragments of SETD2 along with its known domains. **b** Western blot of whole-cell lysates of 293T cells expressing Halo-SETD2 constructs probed with the depicted antibodies. **c**, **e** Microscopy images showing the localization of GFP-SETD2 fragments. The scale bar is 1 mm for 10 × images and 10 µm for the magnified ones. See the text for more details. **d** Western blot of whole-cell lysates of cells shown in **e** probed with the depicted antibodies

We wanted to test whether a specific stretch of the N-terminal region of SETD2 causes its robust degradation. To test that, we made sub-fragments of the N-terminal region, 1–503 (Na) and 504–1403 (Nb), and tested their expression (Fig. 3a). Strikingly, western blotting of whole-cell lysates with an anti-Halo antibody revealed that both the fragments expressed robustly in 293T cells with their expression level similar to SETD2 C; whereas, SETD2 FL could not be detected (Fig. 3b). Notably, microscopy analyses revealed that the fragment Nb was cytoplasmic, unlike Na and C which were nuclear (Fig. 3c). To test whether the localization of this fragment alters its stability, the c-myc **n**uclear **l**ocalization **s**ignal (NLS) was added to GFP-Nb (Nb’) and the expression was checked. Western blotting of the whole-cell lysates revealed a reduced expression level of Nb’ as compared to Nb with continued sensitivity to MG132 treatment (Fig. 3d). Microscopy confirmed that the addition of NLS resulted in the nuclear translocation of Nb and also, reduced its expression level (Fig. 3e). Nevertheless, the expression of all the SETD2 fragments was very robust compared to SETD2 FL and continued to display sensitivity to MG132.

From these experiments, no specific region emerged in the SETD2 protein that is particularly targeted for UPS-mediated decay.

### SETD2 forms nuclear puncta

**Fig. 4:**
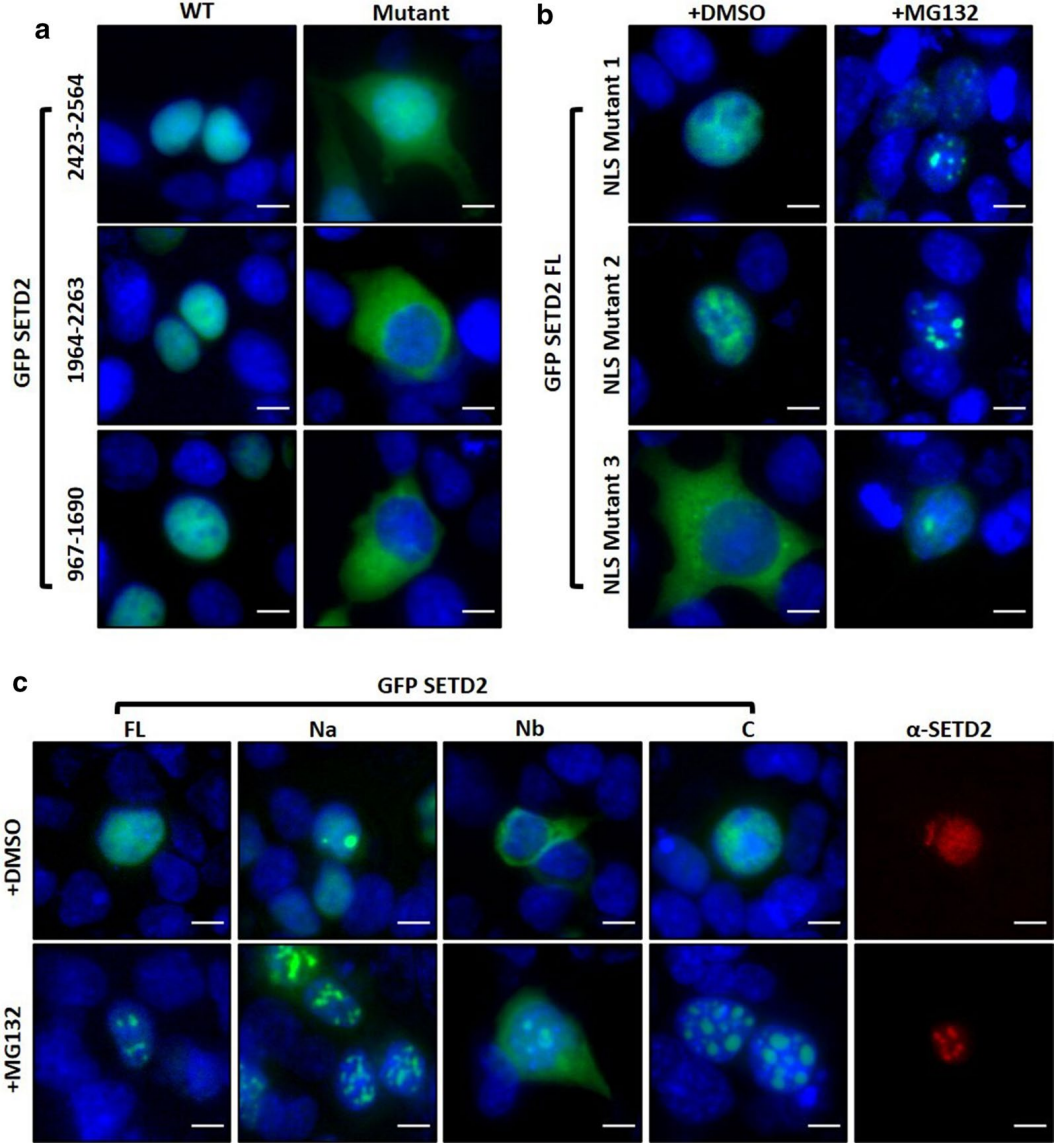
SETD2 forms nuclear puncta. **a**, **b** Microscopy images showing localization of GFP-SETD2 fragments that have putative NLS and the NLS mutants. See the text for more details. **c** Microscopy images showing the localization of SETD2 and its fragments. The scale bar is 10 µm

Microscopy revealed that GFP-SETD2 Na is nuclear and formed puncta (Fig. 3c). This was surprising because this segment lacks a putative NLS with a significant score as per NLS mapper prediction (https://nls-mapper.iab.keio.ac.jp/cgi-bin/NLS_Mapper_form.cgi) [21]. We decided to characterize the NLS of SETD2 to better understand the unexpected localization of the SETD2 fragments. To this end, the localization of a series of GFP-SETD2 fragments was checked using fluorescence microscopy (Additional file 1: Figure S3). Of all the SETD2 fragments tested, the data revealed the presence of putative NLS in three fragments of SETD2: 967–1690, 1964–2263, and 2423–2564 (Fig. 4a). This was consistent with the NLS mapper prediction that revealed the presence of NLS in each of these segments (Additional file 1: Figure S3). To validate these NLS, site-directed mutagenesis was performed to mutate the lysine (K) and arginine (R) residues to alanine (A) and the disruption of the nuclear localization of the mutated GFP-SETD2 fragments was confirmed by microscopy (Fig. 4a, Additional file 1: Figure S3b). Further, to validate that the full-length SETD2 has only these three NLSs, site-directed mutagenesis was performed to mutate these NLS one by one (Fig. 4b). The cytoplasmic localization of the GFP-SETD2 FL mutant, in which all the three NLS were disrupted, confirmed that SETD2 has three NLS (Fig. 4b). Importantly, this also shows that the SETD2 fragment Na forms nuclear puncta without an NLS.

To test whether the full-length SETD2 protein also behaves similarly, the localization of NLS mutants of SETD2 was tested upon MG132 treatment. Strikingly, all the mutants exhibited the formation of nuclear puncta (Fig. 4b). To characterize the aggregate-prone tendency of SETD2 further, 293T cells expressing GFP-SETD2 truncations (described in Fig. 2) were observed under the microscope with or without MG132 treatment. Remarkably, all the SETD2 fragments responded to the treatment with MG132 and demonstrated a marked increase in the formation of puncta (Fig. 4c). Notably, the completely cytoplasmic fragment Nb showed a pan-cellular distribution with a tendency to form puncta upon MG132 treatment similar to SETD2 FL NLS Mutant 3. Furthermore, puncta were also formed by WT GFP-SETD2 FL protein suggesting that it might not be an artifact caused due to protein misfolding resulting from the truncations or mutations (Fig. 4c). To test whether the endogenous SETD2 behaves similarly, immunofluorescence of 293T cells was performed with an anti-SETD2 antibody. Importantly, proteasomal inhibition caused speckle-like staining, revealing that endogenous SETD2 also forms puncta (Fig. 4c). This observation, together with the fact that despite very weak expression, SETD2 FL is aggregate prone suggests that aggregation is an intrinsic property of the SETD2 protein that is exacerbated by an increased protein abundance.

### SETD2 forms ubiquitinated insoluble aggregates

**Fig. 5:**
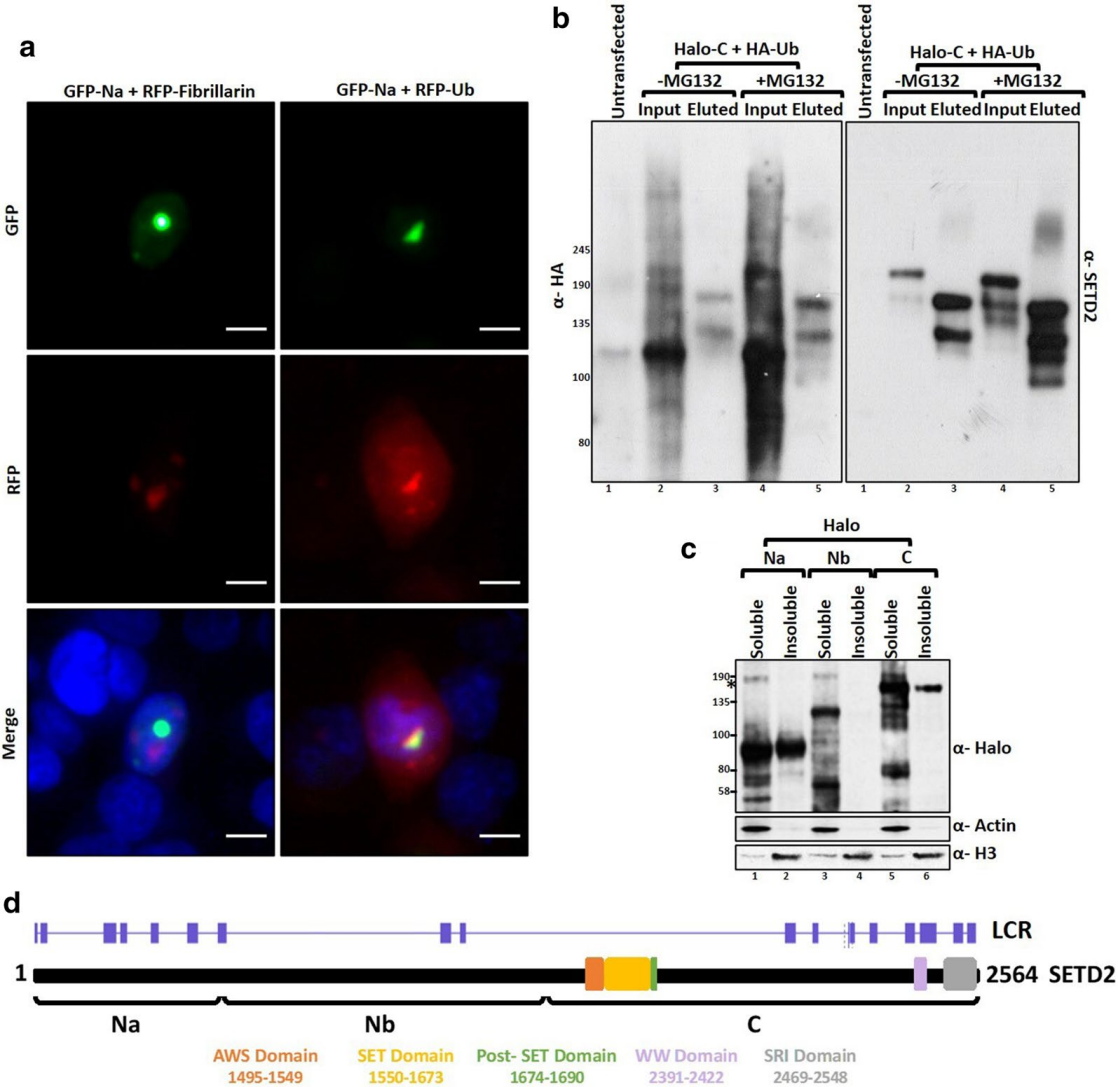
SETD2 forms insoluble ubiquitinated aggregates. **a** Microscopy images showing the colocalization of GFP-SETD2 Na fragment with RFP-Fibrillarin or RFP-Ub. The scale bar is 10 µm. **b** Halo purification was performed from extracts of 293T cells co-expressing Halo-C and HA-Ub. Eluted and 0.5% of Input samples were resolved on a gel and probed with the depicted antibodies. **c** Soluble and insoluble fractions were separated from 293T cells expressing Halo-SETD2 fragments, resolved on a gel, and probed with the depicted antibodies. *Non-specific. **d** Cartoon depicting the predicted low-complexity regions (LCRs) in SETD2. The prediction was performed using the webserver at https://repeat.biol.ucy.ac.cy/fgb2/gbrowse/swissprot/

Exogenously expressed as well as the endogenous SETD2 formed puncta, especially upon MG132 treatment, that are reminiscent of inclusion bodies formed by aggregated proteins. The most striking pattern was observed with the fragment SETD2 Na that formed distinct puncta even in the absence of proteasome inhibition. As aggregates are often ubiquitinated, to confirm that the puncta formed by SETD2 are aggregated structures, the colocalization of RFP-ubiquitin with GFP- SETD2 Na was tested. Clear colocalization was observed suggesting that SETD2 Na puncta are indeed ubiquitinated aggregate structures (Fig. 5a). GFP-SETD2 Na did not colocalize with RFP-Fibrillarin indicating that the puncta were not nucleolar (Fig. 5a). To biochemically substantiate that SETD2 is ubiquitinated, Halo-C was affinity purified from HEK293T cells co-expressing HA-ubiquitin with or without MG132 treatment. The purified proteins were then resolved on a gel and analyzed for the presence of ubiquitination. Western blotting with an anti-HA antibody revealed that SETD2 C was indeed ubiquitinated (Fig. 5b).

To confirm further that SETD2 forms aggregates, we checked the solubility of Halo-SETD2 fragments Na, Nb, and C. 293T cells expressing the Halo-SETD2 fragments were lysed, their soluble and insoluble fractions separated and analyzed by western blotting with an anti-Halo antibody. Correlating with the microscopy observations, SETD2 Na that formed spontaneous puncta was highly insoluble; C was insoluble to a lesser extent (Fig. 5c). The segment Nb was soluble (Fig. 5c). Interestingly, the data show that the different regions of the same protein have different aggregation propensity under similar experimental conditions and are not merely a consequence of expression from a strong CMV promoter. Furthermore, the presence of low-complexity regions (LCR) in a protein is linked to its aggregation propensity [22–24]. Hence, we analyzed the SETD2 protein sequence for the presence of LCR. Strikingly, the solubility profile observed for the SETD2 fragments correlated very well with the LCR distribution of the protein (Fig. 5d).

Collectively, our data show that SETD2 forms aggregated ubiquitinated puncta (discussed in the Discussion section).

### Removal of the N-terminal region of SETD2 leads to a marked increase in global H3K36me3 levels

**Fig. 6:**
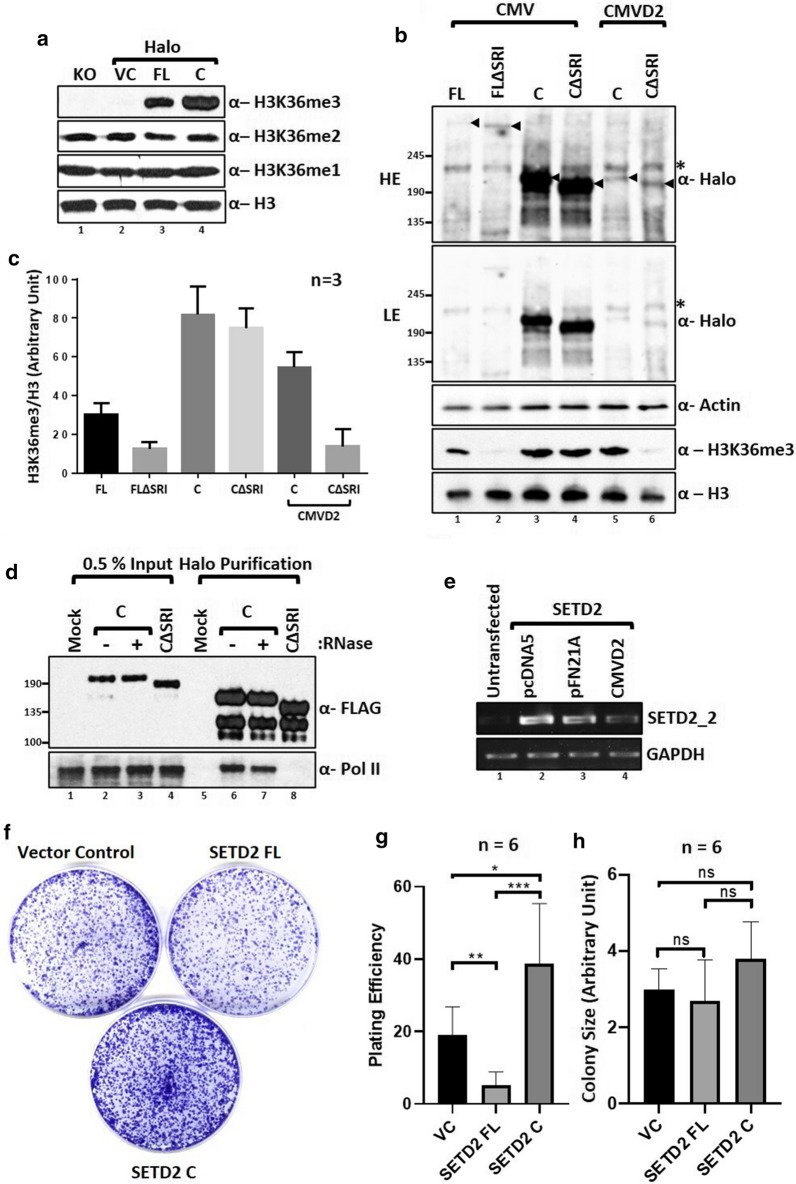
SETD2 has reduced Pol II dependency at high expression levels. **a** Western blot of whole-cell lysates probed with the depicted antibodies. *setd2Δ* 293T (KO) cells were transfected with Halo-vector control (VC), SETD2 full-length (FL) or SETD2 C (C), and the lysates were prepared 72 h post-transfection. **b** Western blot of whole-cell lysates of 293T cells expressing Halo-SETD2 constructs, under the control of either CMV or CMVD2, probed with the depicted antibodies. The expected bands are marked by arrows. *Non-specific. **c** Bar graph showing H3 normalized H3K36me3 signal intensity of data depicted in **b**. The data plotted are from three independent biological replicates. **d** Halo purification was performed from extracts of 293T cells expressing Halo-SETD2 C. Input and eluted samples were resolved on a gel and probed with the depicted antibodies. **e** RNA was isolated from 293T cells expressing SETD2 from different vectors and RT-PCR was performed to check transcript levels. GAPDH was used as a normalization control. **f** Plates showing the results of the colony formation assay to test the effect of introducing full-length SETD2 (FL) or SETD2 C under the control of a CMV promoter on the cell proliferation of *setd2Δ* 293T cells. **g**, **h** Quantification of the number and size of colonies obtained in the colony formation assay (*n* = 6). Unpaired t test was used for statistical analysis. *P* value is < 0.05

We found that the removal of the N-terminal region of SETD2 leads to the stabilization of the remaining portion that shares conserved domains with ySet2. Next, we investigated whether the removal of the N-terminal region affects the catalytic activity of SETD2. To check the activity of the exogenously introduced SETD2, *setd2Δ* (KO) 293T cells were used. Consistent with the role of SETD2 as the sole H3K36me3 depositor in humans, in the KO cells the H3K36me3 mark was not detected in the whole-cell lysates by immunoblotting (Fig. 6a). Next, constructs to express Halo-tagged SETD2 FL or SETD2 C were introduced in the KO cells by transfection. 72 h post-transfection, whole-cell lysates were prepared and analyzed by western blotting. The expression of the empty vector (VC) did not rescue H3K36me3 as expected (Fig. 6a). Strikingly, the expression of SETD2 C in KO cells led to a marked increase in the H3K36me3 level as compared to the rescue with SETD2 FL (Fig. 6a). The other two H3K36 methyl marks, H3K36me1, and H3K36me2, largely remained unchanged (Fig. 6a).

Thus, the removal of the N-terminal segment leads to a marked increase in the global H3K36me3 level. Also, these experiments demonstrate that in the absence of the N-terminal region, SETD2 retains its histone methyltransferase activity.

### At high cellular levels, SETD2 has a reduced RNA pol II dependency for H3K36me3 deposition

Studies conducted in yeast have revealed that the deposition of the H3K36me3 mark is strictly dependent on the ySet2–Pol II association [12]. We were curious whether the marked increase in the global H3K36me3 level upon SETD2 C expression happens in an RNA Pol II-dependent manner.

To test this, Halo-SETD2 constructs without the SRI domain were introduced in *setd2Δ* 293T cells. Similar to the findings for ySet2 in yeast, removal of the SRI domain from full-length SETD2 protein (FLΔSRI) led to a marked decrease in H3K36me3 levels as compared to the FL (Fig. 6b, c). Strikingly though, removal of the SRI domain from SETD2 C (CΔSRI) had a very marginal effect on the H3K36me3 levels (Fig. 6b, c). To confirm that the removal of the SRI domain leads to the abolishment of SETD2–Pol II interaction, Halo-FLAG-SETD2 C and Halo-FLAG-SETD2 CΔSRI were affinity purified from 293T extracts using Halo ligand-conjugated magnetic resin. Elution of proteins purified using this technique involves cleaving the Halo tag with TEV protease, leaving the FLAG epitope which can be detected on the eluted bait by immunoblotting (Fig. 6d). Immunoblotting with an anti-Pol II antibody confirmed that the deletion of the SRI domain from SETD2 leads to the abolishment of SETD2–Pol II interaction (Fig. 6d).

We wondered whether the decreased dependency on Pol II interaction for the H3K36me3 activity of SETD2 C is due to the loss of a possible autoinhibition by the N-terminal region of SETD2 or is due to the increased expression of SETD2 C fragment as compared to the full-length protein. To address these possibilities, Halo-SETD2 C constructs under the control of the CMVD2 promoter were introduced in *setd2Δ* cells. CMVD2 promoter is a truncated form of CMV and exhibits a much weaker transcription activity. The weaker activity of the CMVD2 promoter was confirmed by RT-PCR (Fig. 6e). The reduced expression of SETD2 C under the regulation of CMVD2 promoter was verified by analyzing whole-cell lysate with an anti-Halo antibody (Fig. 6b). Notably, analysis of H3K36me3 revealed that the RNA Pol II dependency of SETD2 C was restored at a reduced expression level as SETD2 CΔSRI did not exhibit much activity when expressed using the CMVD2 promoter (Fig. 6b, c).

SETD2 is considered a tumor suppressor [25]. With this in mind, we tested the effect of SETD2 expression on cell proliferation by introducing full-length SETD2 (FL) or SETD2 C under the control of the CMV promoter in *setd2Δ* 293T cells. Consistent with its association with tumor-suppressive phenotype, the reintroduction of SETD2 FL led to a decrease in cell proliferation (Fig. 6f, g). Strikingly, expression of SETD2 C led to a significant increase in the proliferation of cells as judged by the results of the colony formation assay (Fig. 6f, g). An increase in the colony size was also observed; however, the differences were not statistically significant (Fig. 6h).

We conclude that high cellular levels of SETD2 result in an RNA Pol II-independent H3K36me3 deposition and an increase in cell proliferation.

## Discussion

In recent years, many reports have highlighted the adverse effect of the loss of H3K36me3 that occurs upon SETD2 deletion. Here, we show that the other end of the spectrum can also be detrimental as an excess of SETD2 can lead to inadvertent consequences. Previously, SETD2 has been reported to be regulated by the proteasome [16]. We reveal that the absence of SETD2 proteolysis results in a Pol II-independent H3K36me3 deposition and protein aggregation. Our work illustrates the importance of the N-terminal segment of SETD2, which has been a mystery, in maintaining the requisite intracellular amount of the protein.

### Regulation of SETD2 half-life is important for regulating its function

Altered protein half-life can lead to abnormal development and diseases such as cancer and neurodegeneration [26]. Considering the role of H3K36me3 in a variety of important cellular processes, it is reasonable that regulating the activity of the methyltransferase responsible for the deposition of this mark is important. The previously uncharacterized N-terminal region, which is absent in ySet2, plays an important role in SETD2 regulation. The gain or loss of protein segments may be an important contributor to the degradation rate of proteins during evolution [27]. The differences in half-lives between homologs might be needed to adjust for the differences in the mechanism of deposition of H3K36 methylation. For instance, unlike in yeast where ySet2 performs all three states of methylation of H3K36, SETD2 does not appear to be majorly responsible for me1 and me2 deposition. Therefore, even with a shorter half-life, SETD2 might be able to do the required H3K36me3 deposition. Some evidence for this is provided by studies on human cancers which show that the total H3K36me3 levels are not significantly impacted by a monoallelic loss of SETD2 [28, 29].

### SETD2 over-abundance might have inadvertent consequences

We found that SETD2 is an inherently aggregate-prone protein and its aggregation tendency is positively correlated with the presence of LCRs. Aggregation induced by LCRs can be detrimental and there are more than 20 genetic disorders linked to the expansion of trinucleotide repeats within coding sequences that generates LCRs. The uncontrolled expansion of CAG triplets that leads to polyQ tracts is associated with Huntington disease and several ataxias [30]. Overaccumulation of SETD2 can lead to the formation of stable and insoluble protein aggregates impeding the normal function of other proteins. SETD2 aggregation might lead to the co-aggregation of other proteins leading to their inactivation and proteotoxic stress. Ubiquitinated aggregates like the ones formed by SETD2 can directly inhibit or clog proteasomes [31, 32]. As for aggregation of a protein a concentration threshold is required, possibly the robust degradation mechanism in cells ensures that SETD2 is kept at levels that maintain its solubility and activity. Interestingly, SETD2, aka, HYPB were initially identified in a screen to find interactors of the aggregate-prone protein Huntingtin (Htt) [33]. It is possible that the reported Huntingtin–SETD2 interaction was due to the aggregation propensities of these proteins. Interestingly, the N-terminus of SETD2 (SETD2 Na) behaves very similarly to the polyQ containing N-terminus of Huntingtin protein. Like mutant Htt, SETD2 Na localizes to the nucleus in the absence of an NLS and forms spontaneous puncta characteristic of aggregated proteins [34]. The nucleoplasm promotes the formation of such aberrant and insoluble protein aggregates due to the strong crowding forces from highly concentrated macromolecules (approximately 100 mg/ml) [35].

Instead of impeding cellular activities, aggregates can also play functionally important roles. Aggregates formed by SETD2 may indicate the formation of functional bodies such as the RNA granules. RNA granules are micron-sized membrane-less entities formed by phase separation [36]. RNA granules are complex structures that contain ribosomal subunits, translation factors, scaffold, and RNA-binding proteins. These factors control the stability, localization, and translation of RNA cargo [37]. Notably, RNA Pol II also forms granules [38]. Considering the established interaction between SETD2 and Pol II, there might be an interplay between the granules formed by SETD2 and RNA Pol II. Strikingly, the expression of SETD2 C led to an increase in the proliferation of cells. This is consistent with the previous report that SETD2 depletion caused decreased cell proliferation in 293T and HKC8 cells [9]. Increased accumulation of SETD2 might cause it to phase separate, which may lead to a stabilization of transcripts which, in turn, might induce signaling pathways leading to cell proliferation. This possibility along with the likelihood of SETD2 forming part of RNA granules to regulate transcription and translation will be interesting to investigate in the future.

### Pol II association is required for enhancing SETD2 activity

Our data show that when the expression level of SETD2 is high, it has a reduced dependency on RNA Pol II association for H3K36me3 deposition. Possibly, the interaction with Pol II is required for the activation of SETD2 enzymatic activity. When present in its normal cellular amounts, SETD2 is not active enough to lead to a robust H3K36me3 deposition without the Pol II association. Pol II association enhances SETD2 activity and hence, although SETD2 FL protein is barely detectable, the rescue of H3K36me3 can be readily seen in the *setd2Δ* cells. At high abundance, despite its low activity, a robust H3K36me3 deposition is seen likely due to the increased copy number of the SETD2 protein in cells. Recent studies in yeast have also challenged the notion that the Pol II association is required for chromatin recruitment of Set2 [39]. The study found that the engagement of Set2 and Pol II through the SRI domain is instead required for the activation of Set2. Recent findings highlight the importance of the regulation of SETD2 activity in cells. This report shows that H3.3S31ph can enhance H3K36me3 deposition by augmenting SETD2 activity, leading to rapid, high-level expression of stimulation-induced genes [40]. At a high cellular level, SETD2′s dependency on such factors might be reduced which can lead to unintended transcriptional changes. By keeping the SETD2 expression level low and thus, maintaining its requirement for activation by factors such as H3.3S31ph and Pol II, the cells can regulate H3K36me3 deposition and avoid unintended transcriptional changes. Additionally, the Pol II-independent deposition of H3K36me3 can result in the redistribution of the H3K36me3 mark resulting in the mistargeting of important epigenetic regulators. The PWWP domain-containing proteins DNMT3a, MutSα, and MORF depend on H3K36me3 for proper recruitment [4, 5, 17, 41]. It is important to note that besides histone H3, SETD2 also has non-histone targets like tubulin, the methylation of which is important for the metaphase transition [42]. Hence, the consequences of an increase in SETD2 abundance might not be limited to changes in histone methylation.

## Conclusion

SETD2 is robustly degraded by the proteasome and the removal of its N-terminal region leads to its stabilization. We also discovered that SETD2 is an aggregate-prone protein. We characterized the nuclear localization signals (NLS) in SETD2 and found that strikingly, SETD2 can form nuclear puncta even without an intact NLS. This behavior is very similar to that exhibited by well-characterized aggregate-prone proteins like the mutant huntingtin. Importantly, it suggests that the robust degradation of SETD2 might be a mechanism to prevent inadvertent protein aggregation mediated by SETD2 and of SETD2 itself. This can also be a mechanism to regulate the possible liquid phase transition of SETD2 that needs to be investigated in future studies. Importantly, an increased accumulation of SETD2 also results in a reduced RNA Pol II dependency for H3K36me3 deposition, which is very uncharacteristic. SETD2 has a long N-terminal region that is missing in its yeast homolog Set2. Our work illustrates the importance of this N-terminal segment of SETD2 in maintaining the requisite intracellular amount of the protein.

## Methods

### Plasmids and oligos

SETD2-HaloTag® human ORF in pFN21A was procured from Promega. Deletion mutants of SETD2 were constructed by PCR (Phusion polymerase, NEB) using full-length SETD2 as a template and individual fragments were cloned. All constructs generated were confirmed by sequencing. SETD2-GFP, mRFP-Ub, pLenti puro HA-Ubiquitin, pTagRFP-C1-Fibrillarin and pCDNA3-ySet2 were procured from Addgene. siRNAs were purchased from Dharmacon and oligonucleotides were procured from Integrated DNA Technologies (IDT).

### Cell line maintenance and drug treatment

The cell lines used in this study (HEK293T, HEPG2 and, HELA) were procured from ATCC. Cells were maintained in DMEM supplemented with 10% FBS and 2-mM l-glutamine at 37 °C with 5% CO_2_. MG132 (Sigma) was added at a final concentration of 10 μM for 12 h. Chloroquine (Sigma) treatment was done as indicated in the text. Cycloheximide (Sigma) was added at a final concentration of 10 μg/ml. Transfections were performed at cell confluency of 40% using Fugene HD (Promega) using a ratio of 1:4 of the plasmid (µg) to transfection reagent (µl).

### Colony Formation Assay

The cells (*n* = 4000) were plated in triplicate in 60-mm tissue culture plates and were allowed to grow as a monolayer for 14 days. Cells were incubated in the complete culture medium, with media changes every 2–3 days. After 14 days, the cells were fixed with 4% paraformaldehyde for 1 h. The colonies were stained with 0.5% crystal violet (0.5% in 70% ethanol) for 1 h at room temperature, rinsed and air-dried. Images were captured and the quantification of the number and size of the colonies was done using ImageJ.

### Isolation of total RNA and PCR

Total RNA was extracted from cells as per the manufacturer’s (Qiagen) instructions. It was further treated with DNaseI (NEB) for 30 min at 72 °C to degrade any possible DNA contamination. RNA (2 μg) was subjected to reverse transcription using the QScript cDNA synthesis mix according to the manufacturer’s instructions. cDNAs were then amplified with the corresponding gene-specific primer sets. For RT-PCR, PCR was conducted for 24 cycles using the condition of 30 s at 94 °C, 30 s at 60 °C and 30 s at 72 °C. The PCR products were analyzed on a 1% agarose gels containing 0.5 μg/ml ethidium bromide. The sequence of oligos is in Additional file 1: Figure S4.

### Histone isolation and immunoblot analysis

Histones were isolated and analyzed as described previously [43]. For immunoblotting, histones were resolved on 15% SDS-polyacrylamide gel, transferred to PVDF membrane and probed with antibodies. Signals were detected using the ECL plus detection kit (ThermoFisher).

### Antibodies

H3K36me3 (CST, 4909S), H3K36me2 (Active Motif, 39255), H3K36me1 (Abcam, ab9048), H3 (CST, 9715S), Halo (Promega, G9211), β-actin (Abcam, ab8224), SETD2 (Abclonal, A3194), FLAG (Sigma-Aldrich, A8592), Pol II (Abcam, ab5095).

### Cell Fractionation

To prepare soluble and insoluble extracts, 293T cells were washed with 1xPBS, collected by centrifugation, and resuspended in lysis buffer (50-mM Tris, pH 7.5, 350-mM NaCl, 1% Triton-X 100, 0.1% Na-deoxycholate and a protease inhibitor mix). The lysed cells were centrifuged at 13,000 rpm for 20 min. The supernatant was collected as the soluble fraction. The pellet was washed with lysis buffer containing 600-mM NaCl). The remaining insoluble pellet following another centrifugation was resuspended in Laemmli buffer (Biorad) and solubilized by sonication on ice.

### Affinity purification

293T cells expressing the protein of interest were harvested in 1xPBS and collected by centrifugation. The cells were lysed by resuspending in lysis buffer (50-mM Tris, pH 7.5, 150-mM NaCl, 1% Triton-X 100, 0.1% Na-deoxycholate, and a protease inhibitor cocktail). The lysed cells were centrifuged at 13,000 rpm for 20 min. The supernatant was collected and diluted 1:3 by adding dilution buffer (1× PBS, pH 7.5 with 1-mM DTT and 0.005% NP-40). The diluted lysate was added to 100 µl of pre-equilibrated Magne® HaloTag® Beads (Promega, G7282) and incubated overnight on a rotator at 4 °C. The beads were then washed with wash buffer (50-mM Tris–HCL, pH 7.5, 300-mM NaCl, 0.005% NP-40, and 1-mM DTT. 2 µl AcTEV (ThermoFisher, 12575015) protease was used per 100 µl of elution buffer.

### Immunofluorescence

293T cells were plated onto glass coverslips in a 6-well plate. Cells were washed with 1× PBS and fixed in 4% paraformaldehyde for 20 min at 37 °C. Cells were then washed three times with cold 1 × PBS and permeabilized for 5 min with 1× PBS containing 0.2% Triton-X 100. Permeabilized cells were then blocked for 30 min with blocking buffer (3% BSA and 0.1% Triton-X in 1× PBS). Cells were stained with primary antibodies against SETD2 (1:1,000; Abclonal) for 1 h at room temperature. A secondary antibody conjugated with AlexaFluor 568 was applied for 1 h at room temperature. All the images depicted in the same panel were captured using the same settings.

## Supplementary information

**Additional file 1:** Additional figures.

## Data Availability

All data generated or analyzed during this study are included in this published article and its additional files.

## Abbreviations

PTMs: Post-translational modifications
SRI: Set2–Rpb1 Interaction
AWS: Associated with SET
SET: Su(var)3-9, Enhancer-of-zeste and Trithorax
UPS: Ubiquitin–proteasome system
NLS: Nuclear localization signal
LCR: Low-complexity regions
