# Supplementary material for: Regulation of SETD2 stability is important for the fidelity of H3K36me3 deposition
Source: Epigenetics Chromatin. 2020 Oct 6;13:40. doi: 10.1186/s13072-020-00362-8 (PMC7542105; doi:10.1186/s13072-020-00362-8)
Supplement: Supplementary file 1 — Additional file 1: Additional figures. [file 13072_2020_362_MOESM1_ESM.pdf]

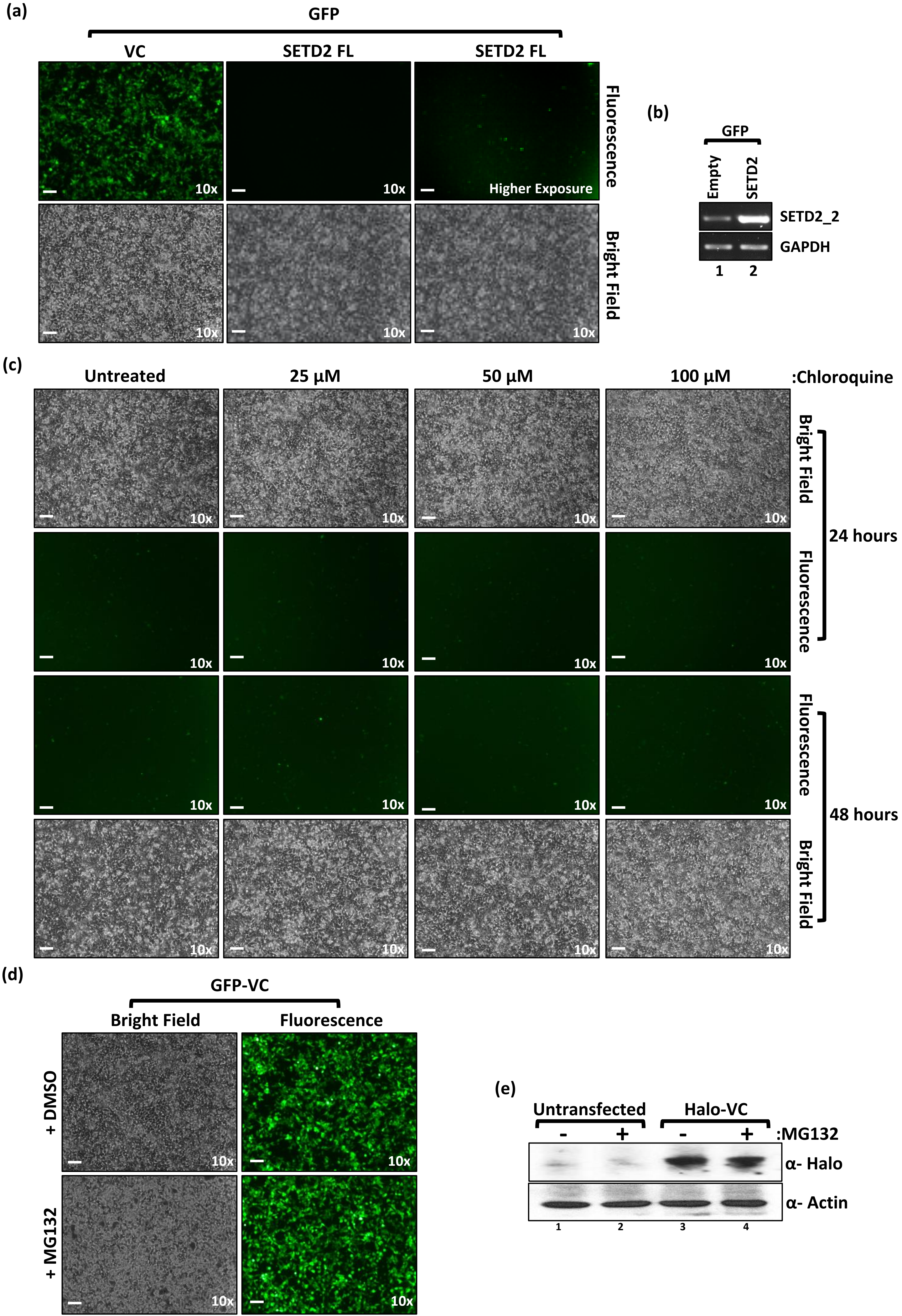

(a)
GFP
VC
SETD2 FL
SETD2 FL
Fluorescence
Bright Field
10x
Higher Exposure
(b)
GFP
Empty
SETD2
SETD2_2
GAPDH
1
2
(c)
Untreated
25 µM
50 µM
100 µM
:Chloroquine
Bright Field
Fluorescence
24 hours
48 hours
(d)
GFP-VC
Bright Field
Fluorescence
+ DMSO
+ MG132
(e)
Untransfected
Halo-VC
- + - +
:MG132
α- Halo
α- Actin
1 2 3 4

Figure S1: (a) Microscopy images showing expression of GFP constructs in 293T cells. The 'Higher Exposure' image is included to more clearly depict expression of the protein. The scale bar in is 1 mm. (b) RNA was isolated from transfected cells described in (a) and RT-PCR was performed to check transcript levels. GAPDH was used as a normalization control. (c) Microscopy images showing the effect of chloroquine treatment on expression of GFP-SETD2 FL in 293T cells. 24 hours post-transfection, chloroquine was added to the culture media at the concentrations and for the time periods depicted. The scale bar in is 1 mm. (d) Microscopy images showing effect of MG132 treatment on expression of GFP in 293T cells. The scale bar in is 1 mm. (e) Western blot of whole-cell lysates probed with the depicted antibodies. Lysates of wild type 293T (untransfected) cells expressing Halo-vector control (VC) were prepared after 12 hrs of MG132 (10 μM) treatment.

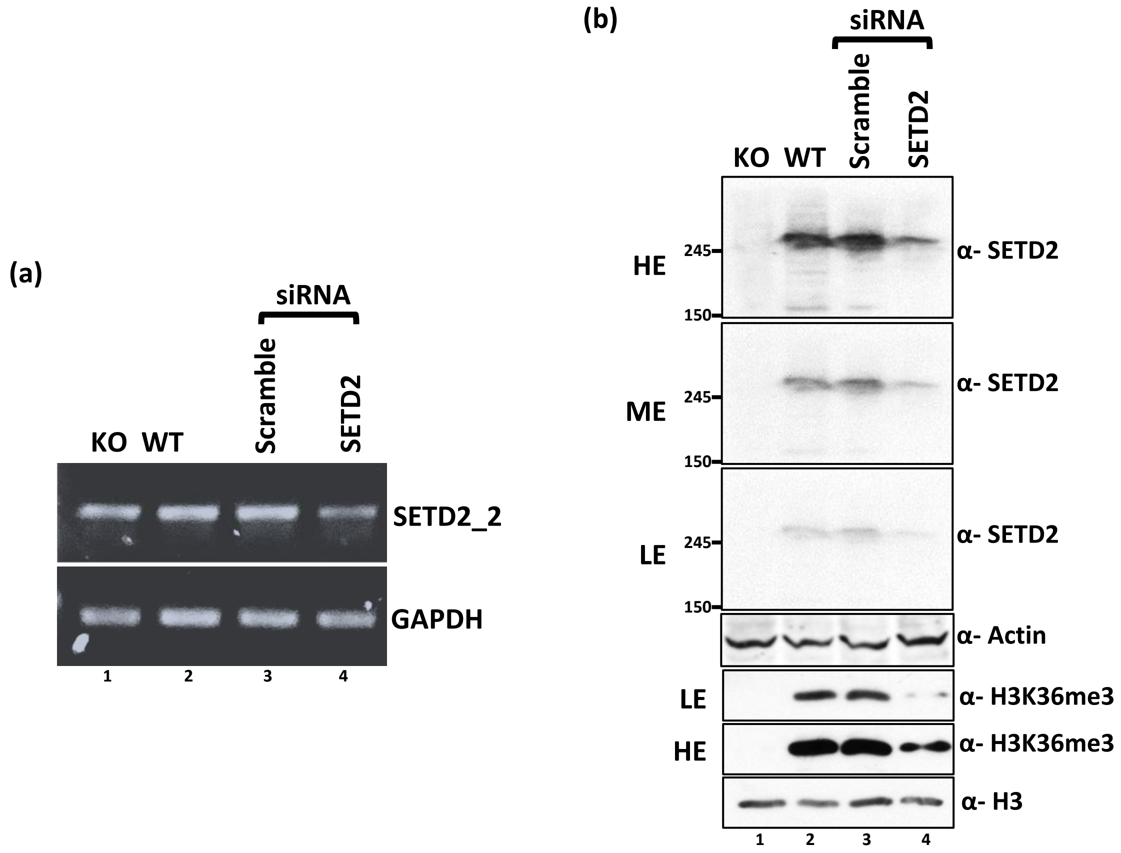

Figure S2: (a) To confirm the specificity of the commercially available SETD2 antibody (from Abclonal) used in this study, we probed lysates from WT, SETD2 KO, SETD2 siRNA and scramble siRNA treated 293T cells. The successful depletion of SETD2 transcripts was first checked by RT-PCR (a)(please note that the KO cells produce SETD2 transcripts in which the open reading frame is disrupted). Next, whole-cell extracts were prepared and probed with an anti-SETD2 antibody. As expected, WT and Scramble lanes exhibited the signal which was depleted in the siRNA SETD2 lysate and not detectable in the KO lane (b). Probing the lysates with an anti-H3K36me3 antibody produced the expected results (b). H3K36me3 was markedly reduced in siRNA SETD2 treated cells and was not detectable in KO cells.

(a)

Predicted monopartite NLS		
Position	Sequence	Score
1400	DRGPLKKRRQEIE	8.5
1401	RGPLKKRRQEI	8.5
1403	PLKKRRQEIE	8

Predicted bipartite NLS		
Position	Sequence	Score
1400	DRGPLKKRRQEIESDSESDGELQDRKKVRVE	6.5
1400	DRGPLKKRRQEIESDSESDGELQDRKKVRVE	5.2
1400	DRGPLKKRRQEIESDSESDGELQDRKKVRVEVE	5.3
1401	RGPLKKRRQEIESDSESDGELQDRKKVRVE	10.1
1401	RGPLKKRRQEIESDSESDGELQDRKKVRVE	6.5
1401	RGPLKKRRQEIESDSESDGELQDRKKVRVEVE	5.7
1401	RGPLKKRRQEIESDSESDGELQDRKKVRVE	5.6
2071	NKEKRKRRSSLSPPSSAYERGTKRPDD	6
2072	KEKRKRRSSLSPPSSAYERGTKRPDD	7.7
2089	ERGTKRPDDRYDTPTSKKKVRIK	5.6
2450	KASKKPKTAEADTSSELAKKSKEVFRKEMS	4.3

(b)

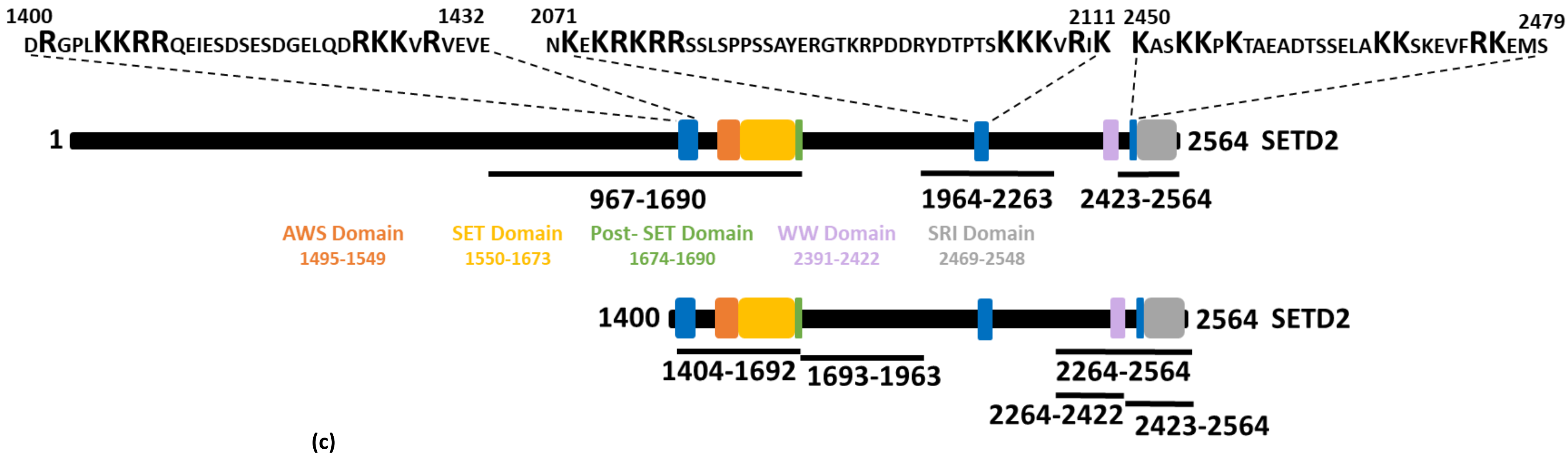

(c)

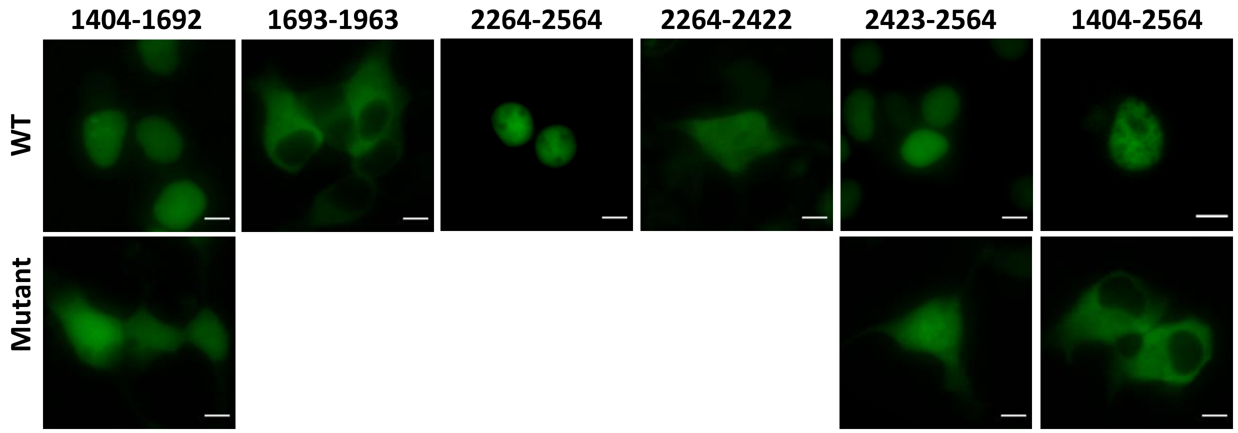

Figure S3: (a) Position, sequence and score of putative NLS in SETD2 based on NLS Mapper prediction. (b) Cartoon illustrating the position and sequence of putative NLS based on NLS Mapper prediction. The highlighted K (lysine) and R (arginine) were mutated to A (alanine) to disrupt NLS in SETD2 fragments 967-1690, 1964-2263 and 2423-2564 as well in the full-length SETD2. The localization of numerous SETD2 fragments were checked before mutating the NLS in the full-length protein. (c) Microscopy images showing localization of SETD2 fragments. The scale bar is 10 µm.

Oligo	Sequence (5’-3’)
SETD2_1_F	GGGAAGGATGAAGAAATTCCAG
SETD2_1_R	CCCTAGATCCTCACTTTTTAAAGG
SETD2_2_F	AAGAAGCTCCCTCTCACCAC
SETD2_2_R	GATCCACATAGGCCTGCATG
GAPDH _F	TTCGACAGTCAGCCGCATCTTCTT
GAPDH _R	CAGGCGCCCAATACGACCAAATC

Figure S4: Sequence of oligos used to perform RT-PCR.
